# Cancer risk in patients hospitalised for Graves’ disease: a population-based cohort study in Sweden

**DOI:** 10.1038/sj.bjc.6605624

**Published:** 2010-03-30

**Authors:** X Shu, J Ji, X Li, J Sundquist, K Sundquist, K Hemminki

**Affiliations:** 1 Center for Family and Community Medicine, Karolinska Institute, 141 83 Huddinge, Sweden; 2 Center for Primary Health Care Research, Lund University, 205 02 Malmö, Sweden; 3 Stanford Prevention Research Center, Stanford University School of Medicine, Stanford, CA 5411, USA; 4 Division of Molecular Genetic Epidemiology, German Cancer Research Center (DKFZ), Im Neuenheimer Feld 580, D-69120 Heidelberg, Germany

**Keywords:** subsequent cancer, hyperthyroidism, Hospital Discharge Registry

## Abstract

**Background:**

The possibility of an association of Graves’ disease (GD) with subsequent cancers raised by certain studies.

**Methods:**

Using a database on 18 156 hospitalised GD patients, subsequent cancers were ascertained.

**Results:**

Increased risks of thyroid and parathyroid tumours were limited to the early follow-up period, which is probably a surveillance bias. Cancer sites with observed excess included the mouth and breast, in contrast to decreased risks of colon cancer, melanoma and non-Hodgkin's lymphoma.

**Conclusion:**

Increased subsequent cancers in GD patients appeared to be balanced by decreased risks at other sites; chance cannot be excluded.

Graves’ disease (GD) is the commonest form of hyperthyroidism, its annual incidence in Sweden ranging from 20 to 30 per 100 000 persons, with an increasing trend in recent years (Abraham-Nordling *et al*, 2008; Stalberg *et al*, 2008) in comparison with approximately 40 per 100 000 in the United States (Stalberg *et al*, 2008). Graves’ disease is four to six times more common in women than in men, with a peak incidence between 20 and 60 years (Brent, 2008; Stalberg *et al*, 2008). It has been suggested that the GD phenotype reflects an interaction between genetic and environmental factors (Brix *et al*, 2001; Ringold *et al*, 2002; Costenbader and Karlson, 2006). Its treatment includes antithyroid drug therapy to reduce the level of thyroid hormone, radioactive iodine I-131 and surgical excision of the thyroid (Brent, 2008; Stalberg *et al*, 2008). In Europe, drug therapy is preferred, while radioiodine and surgery are more often used in the United States (Stalberg *et al*, 2008). Therapy-related side effects include inflammation, agranulocytosis and risk of cancer as response to radioiodine treatment (Franklyn *et al*, 1999; Brent, 2008).

The immune system may be involved in cancer development (de la Cruz-Merino *et al*, 2008; Prestwich *et al*, 2008), as possibly in the dysregulation of the immune system in autoimmune diseases such as GD. Inconsistent associations between GD and malignant tumours have been reported (Cristofanilli *et al*, 2005; Majima *et al*, 2005; Pazaitou-Panayiotou *et al*, 2008). Small study size and variable case reporting weaken the generalisability of the results (Majima *et al*, 2005; Pazaitou-Panayiotou *et al*, 2008). In our cohort study, the largest to date, 18 156 hospitalised GD patients were studied to quantify their subsequent cancer risk.

## MATERIALS AND METHODS

A longitudinal cohort of GD was created by retrieving the details of patients whose last hospitalised thyroid disease was diagnosed as GD between 1964 and 2006 in the nationwide Swedish Hospital Discharge Registry, which is maintained by the National Board of Health and Welfare. We identified GD patients according to the seventh (1964–1968 code 252.0), eighth (1969–86 code 242.0), ninth (1987–96 code 242.0) and tenth (1997–2006 code E05.0) International Classification of Disease codes. This cohort was linked to the latest update (end of 2006) of the Swedish Family-Cancer Database (Hemminki *et al*, 2008; Ji *et al*, 2009). Follow-up for individual risk of malignant tumours was started after the last hospitalisation for GD, and person-years were calculated until diagnosis of cancer, death, emigration or closing date of study, whichever occurred earliest. Considering the concomitant diagnosis of GD and relevant malignant tumours, the first follow-up year was excluded and the risk was reported as All1+ (Hemminki *et al*, 2008; Ji *et al*, 2009). Besides cancers, certain benign tumours, including parathyroid adenomas, are reported to the cancer registry (Nilsson *et al*, 2007; Norenstedt *et al*, 2009). Carcinomas and adenomas were distinguished histologically.

Standardised incidence ratios (SIRs) were calculated as the ratio of observed (O) to expected (E) number of cases. The expected number was calculated for standard incidence rates adjusted for age (5-year groups), sex, period (5-year groups), residential area and socioeconomic status among patients without a history of GD. The analyses were performed using the SAS statistical package v 9.1. (SAS Institute Inc., Cary, NC, USA).

## RESULTS

In this cohort of 18 156 GD patients, a total of 1495 developed subsequent cancer during a median follow-up period of 17 years (0–42 years), giving an overall SIR of 1.13 (95% CI: 1.07–1.19) for the whole follow-up period as shown in Table 1. Only cancer sites with at least 20 cases during the whole follow-up period are listed. The overall SIR among familial cases was 1.66 (*n*=32, 95% CI: 1.14–2.35, data not shown), which was mainly contributed by the increased risk of thyroid gland tumours (*n*=8, SIR=27.21, 95% CI: 11.62–53.88). To minimise the influence of confounding by concomitant diagnoses, cancers diagnosed during the first year after GD were excluded, leaving 1259 cases in the All1+ group; there was no overall significant excess.

As shown in Table 1, both significantly increased and decreased cancer risks after GD were noted. The SIRs were very high for thyroid and other endocrine gland tumours within 1 year of the last hospitalisation for GD; after excluding these cases the risks were not significant. Among 164 thyroid cancers, 89 were categorised as papillary adenocarcinoma, which accounted for 84% of the histologically defined cases available since 1993 (SIR=19.27, 95% CI: 15.47–23.72); among all thyroid cancers in the database, papillary histology accounted for 62%. Other endocrine tumours were histologically dominated by parathyroid adenomas (87%) with an increased risk of 2.55 (*n*=62, 95% CI: 1.96–3.27). The risks of upper aerodigestive tract and breast cancers were significantly increased in both All and All1+, the risk for mouth cancer being significantly increased (SIR=2.30, *n*=9, 95% CI: 1.04–4.38). Significantly decreased risks were observed for colon cancer, melanoma and non-Hodgkin's lymphoma.

In subsequent cancer assay the cohort of 1259 patients in the All1+ category was dominated by women (*n*=1018, male=241, data not shown), who showed a significantly increased risk of breast cancer, whereas the risks for colon cancer and non-Hodgkin's lymphoma were decreased. Only male patients showed an increased risk of thyroid cancer (SIR=5.14).

As a possible predictor of disease severity, we examined cancer risks after GD by the number of hospitalisations. Multiple hospitalisations were associated with an increased risk of breast cancer (SIR=1.29 for ⩾2 hospitalisations *vs* 1.06 for 1 hospitalisation). Late age at last hospitalisation (⩾40 years) was significantly associated with higher risks of lung (SIR=1.36, 95% CI: 1.06–1.71), breast (SIR=1.24, 95% CI: 1.10–1.40) and thyroid (SIR=1.89, 95% CI: 1.00–3.24) cancers (data not shown).

## DISCUSSION

The present study is the largest to date and has the longest follow-up. The overall excess of all cancers (13%) was largely attributable to cancers of the thyroid, other endocrine glands, upper aerodigestive tract and breast. Risks for colon cancer, melanoma and non-Hodgkin's lymphoma were significantly decreased. Studies have shown that all patients with hyperthyroidism or GD in Sweden are hospitalised (Abraham-Nordling *et al*, 2008; Lantz *et al*, 2009). A national coverage with medically diagnosed GD cases in specialist settings provided reliable diagnostic data; limitations include the lack of data on smoking, medication and treatment. Moreover, some associations could be chance findings due to multiple comparisons.

An overall excess of subsequent cancers after GD was limited to the first year of follow-up and no risk was observed in the All1+ category, contrasting with certain reports (Munoz *et al*, 1978; Hancock *et al*, 1995; Franklyn *et al*, 1999). The high overall risk of 6.96 within the first year of hospitalisation may reflect medical surveillance (Hemminki *et al*, 2008; Ji *et al*, 2009). However, for subsequent periods (All1+), the true risk might be underestimated, because the accuracy of diagnosis of cancers was not compromised by lead-time bias: the diagnosis was just shifted to an earlier time.

For thyroid cancer, previous studies have reported inconsistent results (Cole, 1991; Majima *et al*, 2005; Pazaitou-Panayiotou *et al*, 2008). We found increased risks, confined to the early overall follow-up period, though for men the risk was noted even for the All1+ period. The prevalence of occult thyroid tumours has been reported to be about 13% in an autopsy examination; these may remain clinically silent for a lifetime (Angusti *et al*, 2000). The commonest type, papillary adenocarcinoma, is most closely linked to radiation (Delellis *et al*, 2004). Our finding of an increased risk of parathyroid adenoma after GD could be related to surveillance bias or radioiodine treatment, as with thyroid cancer.

An association of breast cancer with thyroid disease is a long-debated issue (Ito and Maruchi, 1975; Smyth *et al*, 1998). Our results on an increased risk of breast cancer, especially for women with multiple hospitalisations, raise the possibility that thyroid hormones play a role in the aetiology of breast carcinoma (Cristofanilli *et al*, 2005). As smoking is a common risk factor for GD and cancers of the lung, upper aerodigestive tract and urinary bladder (Nomura, 1982; Costenbader and Karlson, 2006), the excesses of these cancers could be due to smoking. Among upper aerodigestive tract cancers, mouth cancer risk was increased 2.3 times, in line with an American cohort study, probably indicating treatment effects (Goldman *et al*, 1990).

The deficiencies, particularly among women, of colon cancer, melanoma and non-Hodgkin's lymphoma are novel to our knowledge. These could be caused either by autoimmunity of GD, especially for melanoma and non-Hodgkin's lymphoma, or by pathological thyroid hormone excess (de la Cruz-Merino *et al*, 2008; Prestwich *et al*, 2008). Thyroid-stimulating hormone is a growth factor for melanoma cells, and so low levels of this hormone in GD may suppress melanoma development (Ellerhorst *et al*, 2006).

## Figures and Tables

**Table 1:** SIRs for subsequent cancers in patients hospitalised for Graves’ disease by follow-up time

	**Follow-up interval (years)**																							
	**<1**				**1–4**				**5–9**				**⩾10**				**All**				**All 1+**			
**Cancer site**	**O**	**SIR**	**95% CI**		**O**	**SIR**	**95% CI**		**O**	**SIR**	**95% CI**		**O**	**SIR**	**95% CI**		**O**	**SIR**	**95% CI**		**O**	**SIR**	**95% CI**	
Upper aerodigestive tract	2	3.60	0.34	13.23	4	1.64	0.43	4.23	6	1.76	0.63	3.85	21	1.43	0.88	2.19	33	**1.56**	1.08	2.20	31	**1.51**	1.02	2.14
Stomach	2	3.43	0.32	12.62	0				7	1.76	0.70	3.64	15	0.73	0.41	1.20	24	0.86	0.55	1.28	22	0.81	0.51	1.22
Colon	0				8	0.78	0.33	1.55	11	0.75	0.37	1.34	59	0.81	0.61	1.04	78	**0.78**	0.61	0.97	78	**0.80**	0.63	0.99
Rectum	1	0.87	0.00	4.98	6	1.16	0.42	2.55	5	0.66	0.21	1.56	28	0.75	0.50	1.09	40	0.78	0.56	1.07	39	0.78	0.56	1.07
Liver	0				2	0.94	0.09	3.45	3	0.88	0.17	2.59	23	1.10	0.70	1.66	28	1.04	0.69	1.51	28	1.06	0.70	1.53
Pancreas	1	2.45	0.00	14.02	2	1.02	0.10	3.76	1	0.31	0.00	1.80	21	1.04	0.64	1.60	25	0.97	0.63	1.44	24	0.95	0.61	1.41
Lung	2	1.57	0.15	5.77	13	**2.14**	1.13	3.66	13	1.34	0.71	2.29	60	1.05	0.80	1.35	88	1.19	0.95	1.46	86	1.18	0.94	1.46
Breast	15	1.61	0.90	2.67	49	1.19	0.88	1.57	69	1.20	0.94	1.52	259	1.09	0.96	1.24	392	**1.14**	1.03	1.26	377	**1.12**	1.01	1.24
Cervix	1	0.70	0.00	4.01	3	0.53	0.10	1.57	9	1.39	0.63	2.65	13	0.81	0.43	1.39	26	0.88	0.57	1.29	25	0.89	0.57	1.31
Endometrium	2	1.01	0.10	3.73	7	0.82	0.33	1.70	21	**1.83**	1.13	2.79	41	0.81	0.58	1.09	71	0.97	0.76	1.23	69	0.97	0.76	1.23
Ovary	1	0.73	0.00	4.17	3	0.50	0.10	1.49	6	0.75	0.27	1.64	32	0.96	0.66	1.36	42	0.86	0.62	1.17	41	0.87	0.62	1.18
Prostate	2	1.18	0.11	4.33	7	0.88	0.35	1.83	10	0.79	0.38	1.46	63	0.95	0.73	1.21	82	0.92	0.73	1.15	80	0.92	0.73	1.14
Kidney	2	2.89	0.27	10.61	6	1.95	0.70	4.27	5	1.15	0.36	2.69	21	1.02	0.63	1.56	34	1.18	0.82	1.66	32	1.14	0.78	1.61
Urinary bladder	2	1.76	0.17	6.49	11	**2.19**	1.09	3.93	8	1.11	0.48	2.20	37	1.12	0.79	1.55	58	1.25	0.95	1.62	56	1.24	0.94	1.61
Melanoma	4	2.13	0.55	5.50	4	0.50	0.13	1.29	14	1.34	0.73	2.25	19	**0.53**	0.32	0.82	41	**0.73**	0.52	0.99	37	**0.68**	0.48	0.94
Skin	0				4	0.71	0.18	1.83	11	1.32	0.66	2.37	29	0.70	0.47	1.01	44	0.78	0.57	1.05	44	0.80	0.58	1.07
Nervous system	8	**6.57**	2.81	13.01	6	1.14	0.41	2.50	4	0.57	0.15	1.48	24	0.90	0.58	1.35	42	1.05	0.76	1.42	34	0.88	0.61	1.23
Thyroid gland	145	**234.07**	197.52	275.47	10	**4.04**	1.93	7.46	2	0.70	0.07	2.58	7	0.92	0.36	1.90	164	**12.08**	10.30	14.07	19	1.47	0.88	2.29
																								
Endocrine glands	44	**42.17**	30.63	56.64	7	1.57	0.62	3.25	8	1.37	0.58	2.71	12	0.57	0.29	1.00	71	**2.20**	1.71	2.77	27	0.86	0.57	1.26
Parathyroid	*42*	** *50.80* **	*36.60*	*68.71*	*5*	*1.45*	*0.46*	*3.42*	*5*	*1.14*	*0.36*	*2.68*	*10*	*0.65*	*0.31*	*1.20*	*62*	** *2.55* **	*1.96*	*3.27*	*20*	*0.86*	*0.53*	*1.33*
Non-Hodgkin's lymphoma	1	1.09	0.00	6.25	2	0.48	0.05	1.78	5	0.83	0.26	1.95	18	**0.62**	0.36	0.98	26	**0.64**	0.42	0.95	25	**0.63**	0.41	0.94
Leukaemia	0				1	0.31	0.00	1.76	4	0.84	0.22	2.18	22	0.96	0.60	1.45	27	0.85	0.56	1.24	27	0.87	0.57	1.27
All	236	**6.96**	6.10	7.91	162	1.08	0.92	1.26	233	1.11	0.97	1.26	864	**0.93**	0.87	0.99	1495	**1.13**	1.07	1.19	1259	0.97	0.92	1.03

Abbreviations: O=observed; SIR=standardised incidence ratio; CI=confidence interval.

Bold type, 95% CI does not include 1.00; bold type with underline, 99% CI does not include 1.00.
